# Turkish Pediatric Rheumatology Society consensus statements on systemic onset juvenile idiopathic arthritis in Turkey

**DOI:** 10.1186/1546-0096-13-S1-P134

**Published:** 2015-09-28

**Authors:** B Sozeri, N Aktay Ayaz, S Turgay, B Makay, E Demirkaya, E Unsal, O Kasapcopur, S Ozen

**Affiliations:** 1 Erciyes University Faculty of Medicine, Pediatric Rheumatology, Kayseri, Turkey; 2 Istanbul Kanuni Sultan Suleyman Education and Research Hospital, Pediatric Rheumatology, Istanbul, Turkey; 3 Novartis Pharma, Medical Department, Istanbul, Turkey, Istanbul, Turkey; 4 Dokuz Eylul University Faculty of Medicine, Pediatric Rheumatology, Izmir, Turkey; 5 Gulhane Military Medical Faculty, FMF Arthritis Vasculitis and Orphan disease Research Center, Ankara, Turkey; 6 Cerrahpasa Medical Faculty, Istanbul University, Pediatric Rheumatology, Istanbul, Turkey; 7 Hacettepe University Faculty of Medicine, Pediatric Rheumatology, Ankara, Turkey

## Objective

The aim of this study was to describe the current practice and to reach consensus of management in patients with systemic juvenile idiopathic arthritis (sJIA) in the Turkey.

## Methods

Recommendations were established via consensus by a panel of experts in pediatric rheumatology, based on analysis of available scientific evidence obtained from the clinical experience of panelists. The Delphi method solicits the opinion of an expert panel through a carefully designed questionnaire which in this case included questions on: epidemiology, diagnosis, treatment of symptoms, drug choice, adverse events, follow-up visits, hospital and emergency service admissions. The responses were analyzed and discussed in a face to face meeting followed by consensus building steps.

## Results

Profiles of sJIA in Turkey were evaluated based on clinical experience of panelist. The percentage of patients diagnosed with JIA was found 0,004% in the general population in Turkey. Estimated number of JIA patients was reported 342 per year. Twenty of them were diagnosed sJIA. Estimated sJIA patients were 55 per year. The median age of diagnosis was 5.5 years. Consensus was achieved on diagnosis of the patients which established by detail investigation inpatients clinics. Both infectious and malignancy must be excluded in the patients before diagnosis.

The all sJIA patients was distributed according to subtypes; monocyclic 23%, polycyclic 30% and persistent/ polyarticular 47%. The mortality rate of sJIA patients was found 1%. The most used drugs were reported as glucocorticoid (100%), methotrexate (58%) and cyclosporine (10%), respectively. Etoposide and IVIG were used only selected patients. In the treatment of disease was determined to be the most preferred drug anakinra after failure DMARDs therapies. Also, we determine that canakinumab and tosilizumab were equally preferred (15-20%). The efficacy of the three drugs was expressed as the same (80-90%). The selection criteria of biological agents used in the treatment were reported: joint involvement was evident, the presence of MAS, characteristics of the patient, effective availability and easy accessibility. The total duration of the treatment was reported two years. The remission rates have been reported to according to the course of the disease; monophasic disease recovered as 100% while polycyclic and poliarticular forms' remission rate were reported 80% and 60%. The morbidity of polyarticular JIA has been reported as high such as joint deformities, growth retardation, delayed puberty, and osteoporosis.

## Conclusions

This consensus, produced through a modified Delphi process, reflects our current recommendations for the diagnosis and management of sJIA in Turkey. The consensus statements are intended to serve as a reference point for teaching, clinical practice, and research in Turkey.

